# A systematic review and recommendations for prom instruments for older people with frailty in emergency care

**DOI:** 10.1186/s41687-022-00438-x

**Published:** 2022-04-01

**Authors:** James David van Oppen, Abdullah Alshibani, Timothy John Coats, Blair Graham, Patricia Holch, Jagruti Lalseta, Nicola Mackintosh, Vivien Richardson, Peter Riley, Jose M. Valderas, Simon Paul Conroy

**Affiliations:** 1 Department of Health Sciences, University of Leicester, Leicester, UK; 2 Emergency and Specialist Medicine, University Hospitals Leicester NHS Trust, Leicester, UK; 3 Emergency Medical Services Department, King Saud Bin Abdulaziz University for Health Sciences, Riyadh, Saudi Arabia; 4 Department of Cardiovascular Sciences, University of Leicester, Leicester, UK; 5 University of Plymouth, Plymouth, UK; 6 Leeds Beckett University, Leeds, UK; 7 Leicester, Leicestershire and Rutland Older Persons Patient and Public Involvement Forum, Leicester, UK; 8 Department of Family Medicine, National University Health System, Singapore, Singapore; 9 MRC Unit for Lifelong Health and Ageing, University College London, London, UK

**Keywords:** Frailty, Geriatrics, Emergency medicine, Patient-reported outcome measure, Patient satisfaction, Person-centred care

## Abstract

**Introduction:**

The current service metrics used to evaluate quality in emergency care do not account for specific healthcare outcome goals for older people living with frailty. These have previously been classified under themes of ‘Autonomy’ and ‘Functioning’. There is no person-reported outcome measure (PROM) for older people with frailty and emergency care needs. This study aimed to identify and co-produce recommendations for instruments potentially suitable for use in this population.

**Methods:**

In this systematic review, we searched six databases for PROMs used between 2010 and 2021 by older people living with frailty receiving acute hospital care. Studies were reviewed against predefined eligibility criteria and appraised for quality using the COSMIN Risk of Bias checklist. Data were extracted to map instrument constructs against an existing framework of acute healthcare outcome goals. Instrument face and content validity were assessed by lay collaborators. Recommendations for instruments with potential emergency care suitability were formed through co-production.

**Results:**

Of 9392 unique citations screened, we appraised the full texts of 158 studies. Nine studies were identified, evaluating nine PROMs. Quality of included studies ranged from ‘doubtful’ to ‘very good’. Most instruments had strong evidence for measurement properties. PROMs mainly assessed ‘Functioning’ constructs, with limited coverage of ‘Autonomy’. Five instruments were considered too burdensome for the emergency care setting or too specific for older people living with frailty.

**Conclusions:**

Four PROMs were recommended as potentially suitable for further validation with older people with frailty and emergency care needs: COOP/WONCA charts, EuroQol, McGill Quality of Life (Expanded), and Palliative care Outcome Scale.

**Supplementary Information:**

The online version contains supplementary material available at 10.1186/s41687-022-00438-x.

## Introduction

Research and quality improvement in emergency care usually focus on service metrics such as length of stay and readmission rates, which do not necessarily align with patient perspectives [1, 2]. Metrics such as mortality and length of stay are convenient as they are easily generated, but may not be meaningful to older people with frailty [3, 4].

**Fig. 1:**
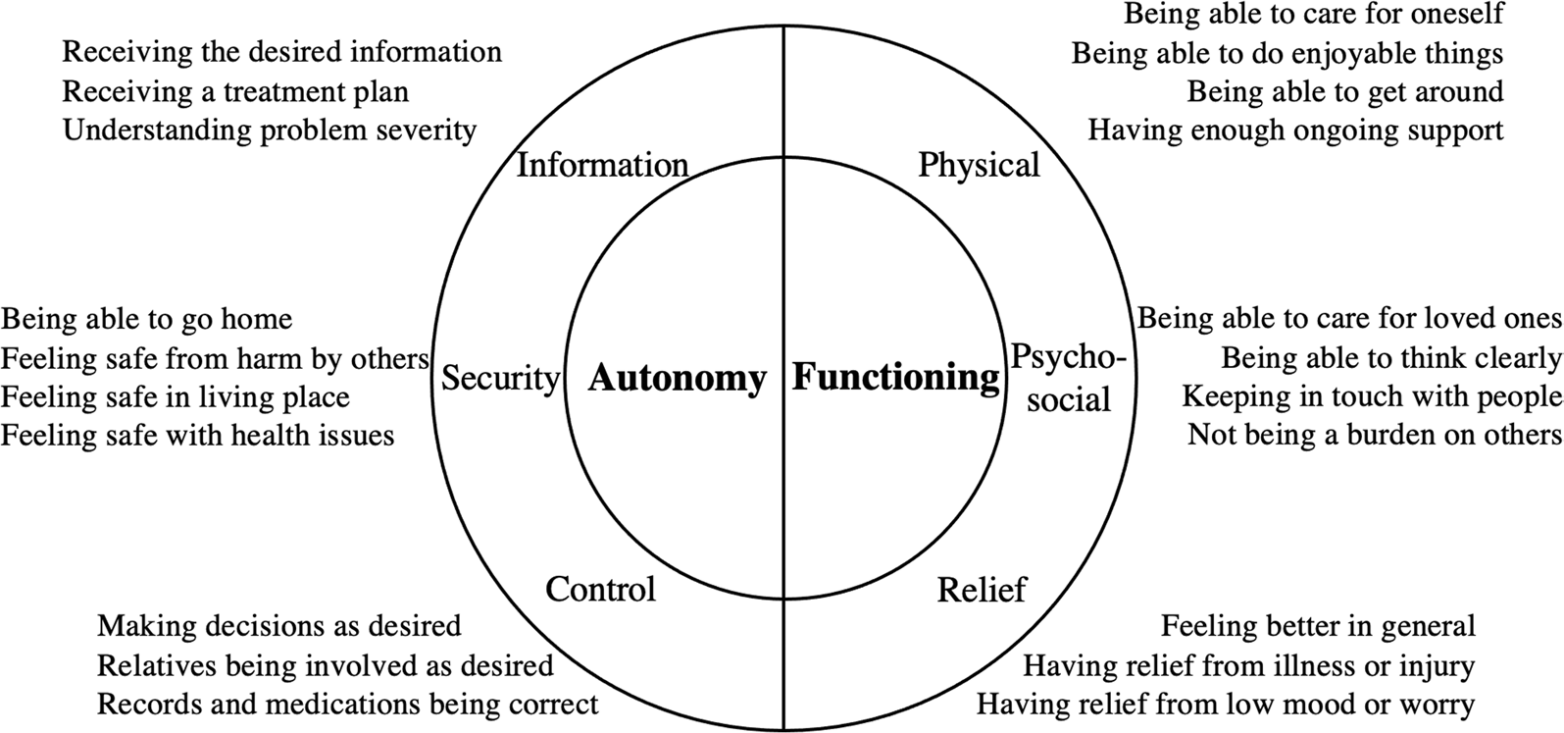
Acute healthcare outcome goals for older people living with frailty classified in qualitative research [5]

We have previously classified meaningful healthcare outcome goals for acute care among older people living with frailty by themes of *autonomy* and *functioning* based on empirical qualitative research with this target population (Fig. 1) [5]. Autonomythemed goals include information (knowing what the problem is), security (being able to live without fear), and control (being able to make healthcare decisions). Functioning-themed goals include physical (being able to self-care and move), psychosocial (being able to think and interact with others), and relief (feeling better from the health problem). The predominance of person-centredness themes across the outcome goals reiterates the vulnerability of older people with frailty in the acute care setting [4].

The attainment of healthcare outcome goals should be ascertained directly from patients themselves. This is possible using Patient-Reported Outcome Measures (PROMs) and Patient-Reported Experience Measures (PREMs) [6, 7]. By capturing data to monitor and compare individuals’ healthcare outcomes, PROMs can measure care effectiveness and contribute to its improvement [8]. The use of PROMs is advocated within the International Federation for Emergency Medicine’s recently updated framework on quality and safety [9]. PREMs consider the process rather than the outcome of healthcare delivery. The framework in Fig. 1 includes concepts measurable using both PROMs and PREMs. This review focuses on the PROMs which best overlap with acute healthcare outcome goals as measures of the effect of that care.

Across the globe, older people represent an increasing proportion of emergency attendances and hospital admissions. There is wide recognition of the need to deliver person-centred holistic care to this group, but the quality of this is poorly captured using existing service metrics [10]. There is currently no consensus about how best to measure emergency care outcomes from older people with frailty [11]. To date, generic PROM instruments have been used to report person-centred emergency care outcomes, but these have been evaluated mainly among younger people, and do not account for either the specific goals of people living with frailty or for the inherent challenges in collecting PROMs in busy emergency care environments [12]. Although there are a number of available instruments that could be used for older people with frailty there is little evidence on their performance in the emergency setting [8].

This systematic review aimed to inform future developments towards more meaningful evaluation of emergency care services for older people living with frailty. The study objectives were to: (1) identify and appraise evidence for existing PROM instruments used to measure outcomes for older people with frailty in other acute hospital settings, and (2) co-produce recommendations for instruments suitable for future emergency care validation studies.

## Methods

### Search strategy

The British Nursing Index (BNI), Cumulative Index to Nursing and Allied Health Literature (CINAHL), Cochrane Database of Systematic Reviews, Embase, Medline, and PsycInfo databases were searched for instruments used to evaluate the outcomes of acute care in hospital for people aged over 65 living with frailty. The strategy searched for instruments studied since 2010, as those in more recent use should have measured concepts relevant to the present-day healthcare system attitudes, processes, and hospital capacity. Emergency care worldwide has been rapidly developing, both in high- and low-income countries. The spectrum of expertise, disciplines involved in the emergency care team, and patients’ case mix and age demographic has been changing. An arbitrary cut-off of 2010 was chosen as a compromise between completeness and relevance to modern patient outcomes of emergency care.

Search strategies using exploded Medical Subject Headings (MeSH) and keywords were adapted from recent literature, and iteratively piloted and developed [13]. Search terms in three blocks (population, setting and intervention) were modified for each database. The detailed search strategy is available in Additional file 1. The review was conducted with reference to the PRISMA-P checklist for preferred reporting and the study protocol was deposited in an open-source registry [14, 15].

Terms relating to Patient-Reported Experience Measures (PREMs) were included in the search strategy. While not seeking to report on PREMs in this review, we included these search terms to limit the likelihood of omitting PROMs and to improve confidence that all relevant literature was identified.

Databases were searched to 24 June 2021. Searches were restricted to English language. Citations were downloaded for management using EndNote software and duplicate articles were removed [16].

### Eligibility

Studies were included which reported the use of PROM(s) in hospital settings by older people living with frailty to measure health-related quality of life or healthcare outcomes from acute, emergency, or urgent care. Multidimensional PROMs were included as frailty affects a heterogenous population who have wide-ranging health conditions associated with multifaceted problems and outcome goals. All citation titles and abstracts were screened by the first author against pre-defined inclusion criteria (Table 1). A 20% sample was screened by the second author. Full texts of identified citations were then downloaded, read, and assessed for eligibility by both reviewers. Cohen’s *kappa* statistic was calculated for interrater reliability for screening decisions. Disagreements were all resolved by discussion. Reasons were recorded for exclusion of ineligible articles at the full text stage. The reference lists of included full texts were hand-searched for additional papers.

**Table 1:** Eligibility criteria

	Include	Exclude
Population	Participants aged over 65 with frailty (include ‘older’, ‘elderly’, ‘senior’, ‘geriatric’) Receiving care in inpatient hospital settings	Younger participants People without frailty Community settings Hospice or rehabilitation settings Outpatient clinics
Intervention	Patient-reported outcome measure	
Outcome	Multidimensional health-related quality of life Acute, emergency, or urgent healthcare outcomes	
Publication	2010 onwards English language Peer-reviewed	Conference abstracts where no full paper has been published

### Appraisal of identified instruments

#### Data extraction and quality assessment

Data from each eligible full text were extracted into a standardised Microsoft Excel form, including study design and setting, psychometric and feasibility outcomes, and themes investigated [17]. Studies were appraised for methodological quality using the COSMIN Risk of Bias checklist [18]. This method guides comprehensive appraisal of PROM development, content and structural validity, reliability and responsiveness, and hypothesis testing. The checklist requires measurement properties to be rated from ‘inadequate’, ‘doubtful’, ‘adequate’, to ‘very good’, counting the worst score. Quality was assessed for each paper and compiled for each instrument. The level of evidence for each measurement property considered the quality and number of studies for each instrument (Table 2). This approach to quality appraisal was adapted from the COSMIN Risk of Bias manual and from a systematic review of studies using an empathy scale [19, 20]. No instruments were excluded based only on quality assessment [20].

**Table 2:** Level of evidence rating criteria

Level	Rating	Criteria
Strong	+ or −	Consistent findings in multiple studies of good methodological quality OR in one study of excellent methodological quality
Moderate	+ or −	Consistent findings in multiple studies of fair methodological quality OR in one study of good methodological quality
Limited	+ or −	One study of fair methodological quality
Conflicting	+ or −	Conflicting findings
Unknown	?	Only studies of poor methodological quality or no studies

+ : positive rating, ?: indeterminate rating, −: negative rating

#### Construct mapping

Each identified PROM was sourced from original development study manuscripts or downloaded from registry websites. The component items and reference time frames, scales, and anchor descriptions were tabulated using Microsoft Excel [17]. Items were categorised by their underlying constructs, as originally defined. To aid recognition of crossover between instrument constructs, closely related categories (such as “Social” and “Social Function”) were merged following discussion with the lay research partners.

The merged instrument constructs were then mapped against the previously established framework of acute healthcare outcome goals for older people living with frailty (Fig. 1) [5].

#### Assessment of face and content validity

A panel of research collaborators was consulted on the potential suitability of the identified PROMs for emergency care. These research collaborators were all members of a Patient and Public Involvement (PPI) Forum specialising in ageing-related research. They included nine lay members who were older people with frailty or their carers and who had personal experience of emergency care. There were also nine professional members who included clinicians and university researchers with expertise in geriatric emergency care.

In an open PPI Forum meeting, the lay and professional collaborators were invited to reflect on the framework of healthcare outcome goals and consider each identified PROM. Feedback was recorded. Each instrument was appraised for face validity and appropriateness for collection from older people with frailty receiving emergency care. Face validity was evaluated by considering whether each instrument relevantly and comprehensively assessed the themes of importance [21, 22]. Instruments which had unanimous consensus for poor face validity or appropriateness to emergency care settings were discarded.

The remaining instruments with potential face validity were then evaluated for their content validity. During a second open PPI Forum meeting, each instrument was presented for discussion in closer detail with the lay and professional collaborators. Discussions focused on accessibility for completion, instrument comprehensiveness, and relevance to older people’s emergency care. Feedback was captured in meeting notes and ensuing emails and was summarised and tabulated for each instrument. Instruments were excluded where there was unanimous consensus for poor content validity.

### Co-production of recommendations

Three of the PPI Forum lay members were recruited as *lay research partners* for further detailed instrument appraisal. Recommendations for potentially suitable instruments were formed through a co-production process with these lay research partners [23]. The lay research partners were consulted separately on four further occasions, with stepwise progress towards recommendations. The first author assumed overall responsibility for the decisions.

In follow-up consultations after each open Forum meeting, the lay research partners read each remaining instrument in full and considered feedback from their peers. Session notes were shared among the lay research partners to ensure accuracy and promote further discussion. All available evidence for methodology quality grades, the construct map, and assessments of face and content validity were reviewed with the lay research partners. Instruments judged as accessibly measuring relevant themes were recommended as suitable for future validation studies.

## Results

**Fig. 2:**
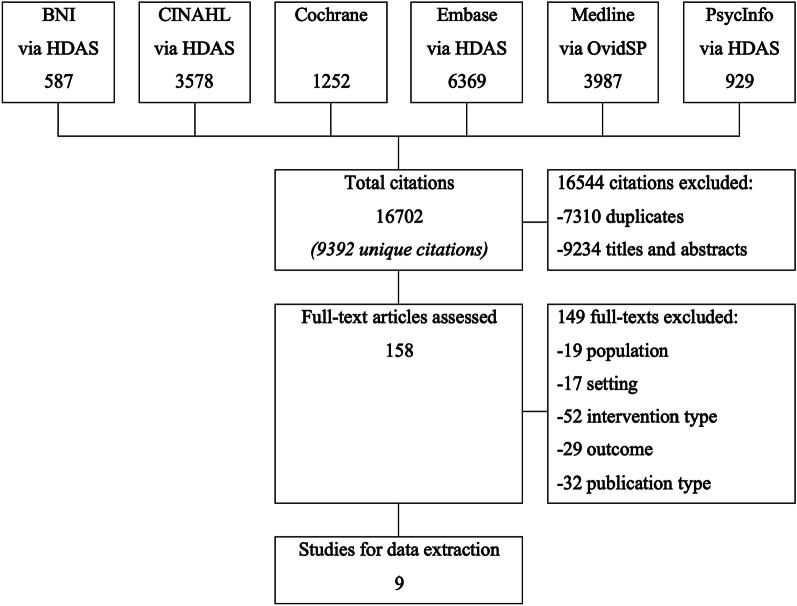
Citation screening for searches to 24 June 2021

After removal of duplicates, 9392 citations were screened (Fig. 2). During title and abstract screening, 9234 citations were excluded. One hundred and fifty-eight full texts were read. Of these, nineteen were excluded for focusing upon younger populations and seventeen for non-hospital settings. Fifty-two studies did not use PROMs, and instead used PREMs, clinical risk tools, or diagnostic instruments. Twenty-nine studies did not address multi-dimensional health-related quality of life or outcomes from healthcare interventions, and thirty-two citations (predominantly conference proceedings) had ineligible publication type or insufficient data for extraction and appraisal. Cohen’s *kappa* statistic for interrater reliability within the 20% double-screened citations showed substantial agreement (*k* = 0.75).

Nine papers evaluated nine instruments used in acute hospital settings to report multi-dimensional outcomes: the COOP/WONCA chart, Duke Health Profile, EuroQol (EQ-5D-3L and EQ-5D-5L were considered together for purposes of suitability selection), the McGill Quality of Life Questionnaire (Expanded) (MQoL-E), Medical Outcomes Study short form survey (SF-36), the Palliative care Outcome Scale (PcOS), Quality of Life in Alzheimer’s Disease (QoL-AD), Readiness for Hospital Discharge Scale for Older People (RHDS-OP-SF), and The Older Persons and Informal Caregivers Survey (TOPICS-CEP) Composite End Point. The data extraction sheet is available as Additional file 2.

Studies reported development of novel or adapted instruments for people receiving palliative care in Canada [24] and for older people being discharged from hospitals in the USA, Ireland, and Switzerland [25]. Other studies from the UK [26], Netherlands [27], and France [28] evaluated instruments for conceptual overlap and convergent validity, while interrater comparison or outcome prediction studies were performed in Australia [29], UK [30], Sweden [31] and Taiwan [32]. Although developed as a condition-specific measure, the QoL-AD was included at this stage as one study examined its collection from participants who did not necessarily have cognitive impairment [31]. Participants’ mean age ranged from 69 to 85 years, with 102 to 17,603 participants included.

Mean instrument length was 18 items, ranging from 5 (EQ-5D) to 42 (TOPICS-CEP). Respondent burden was characterised by response rate in two studies [28, 30] ranging from 50% (EQ-5D) to 73% (Duke), retest response rate (78%, COOP/WONCA) in one study [29] and by data integrity in one further study [27] in which 89% of TOPICS-CEP completions had no missing values. Two studies evaluating QoL-AD [30, 31] both found completion by proxies to underestimate quality of life as reported by patients themselves. QoL-AD was difficult for people with moderate and severe dementia to complete. Most instruments used a reference time period of 0–3 days, while three measured quality of life during the previous week (Duke), fortnight (COOP/WONCA), or month (SF-36).

### Evidence considered and outcomes of recommendation co-production

#### Methodological quality

COSMIN Risk of Bias ratings ranged from ‘Doubtful’ to ‘Very good’ (Table 3). None of the instruments had been evaluated for reliability or serial measurement properties in the setting of interest, and likewise no results were reported for cross-cultural adaptation. The full scoring ratings are tabulated in Additional file 3. One study which developed new instrument items was assigned a moderate score (‘adequate’) for its cognitive interview component [24]. The five studies which evaluated structural validity (usually with factor analysis) and internal consistency (usually with Cronbach’s *alpha* statistic) were all assigned ‘adequate’ or ‘very good’ methodological quality ratings [24–26, 28, 31].

**Table 3:** COSMIN Risk of Bias assessment of studies and level of evidence for measurement properties

Instrument	Paper(s)	Participants	Assessed measurement properties (lowest *COSMIN* rating)							Level of evidence
			Development		Factors		Testing			
			PROM design	Content validity	Structural validity	Internal consistency	Criterion validity	Construct validity	Responsiveness	
COOP/WONCA	Franke (2021)	118	–	–	–	–	–	–	D	Limited (+)
Duke	Perret-Guillaume (2010)	701	–	–	–	V	–	V	–	Strong (+)
EQ-5D-3L	Dzingina (2017)	783	–	–	A	–	–	V	–	Strong (+)
	Lin (2020)	102	–	–	–	–	–	V	–	
EQ-5D-5L	Sheehan (2012)	109	–	–	–	–	–	V	–	Strong (+)
MQoL-E	Cohen (2019)	803	A	A	V	V	–	–	–	Strong (+)
PcOS	Dzingina (2017)	783	–	–	A	–	–	V	–	Strong (+)
QoL-AD	Sheehan (2012)	109	–	–	–	–	–	V	–	Strong (+)
	Torisson (2016)	199	–	–	A	V	–	V	–	
RHDS-OP-SF	Mabire (2015)	998	–	–	V	V	V	–	–	Strong (+)
SF-36	Perret-Guillaume (2010)	701	–	–	–	V	–	V	–	Strong (+)
TOPICS-CEP	Hofman (2017)	17,603	–	–	–	–	–	V	–	Strong (+)

Properties: V: very good, A: adequate, D: doubtful. Evidence: + : positive rating. Instruments: MQoL-E (McGill Quality of Life Questionnaire Expanded), PcOS (Palliative care Outcome Scale). QoL-AD (Quality of Life in Alzheimer’s Disease), RHDS-OP-SF (Readiness for Hospital Discharge Scale for Older People), TOPICS-CEP (The Older Persons and Informal Caregivers Survey composite endpoint)

No instruments had been evaluated for cross-cultural validity, reliability, or measurement error

Most studies evaluated instruments for criterion or construct validity. Methodological quality for these was rated as ‘Very good’, with most studies using regression techniques. In one study investigating COOP/WONCA responsiveness during and following hospital admission, quality was limited by the absence of correlation statistics [29].

The levels of evidence for instrument quality ratings are also summarised in Table 3. Most instruments were graded as having ‘Strong’ positive evidence, having at least one measurement property with ‘Very good’ methodology in at least one study. There was limited evidence supporting the COOP/WONCA chart, as the only investigating study was rated as having ‘Doubtful’ quality.

#### Construct map

The map of instrument constructs to themes of acute healthcare outcome goals is shown in Table 4. Functioning outcomes were the predominant focus in most identified PROMs. Autonomy outcomes were considered in fewer PROMs. All themes in the classification were represented but were not measured comprehensively by any single instrument.

**Table 4:** Instrument constructs mapped against acute healthcare outcome goals for older people living with frailty

Construct	Instrument								
	COOP/WONCA	Duke	EuroQol	MQoL-E	PcOS	QoL-AD	RHDS-OP-SF	SF-36	TOPICS-CEP
Autonomy									
Control	–	–	–	•	•	–	–	–	–
Information	–	–	–	•	–	–	•	–	–
Security	–	–	–	–	–	•	–	•	•
Functioning									
Physical	•	•	•	•	•	•	•	•	•
Psychosocial	•	•	•	•	•	•	–	•	•
Relief	•	•	•	•	•	•	–	•	•

The acute healthcare outcome goals were defined and classified in previous qualitative work [5]

Communication effectiveness, particularly around healthcare information and decision-making, was considered in the MQoL-E and RHDS-OP-SF instruments but not in others. Three instruments measured a ‘self-esteem’ construct, which had some overlap with the ‘security’ and ‘psychosocial’ themes. Two instruments included ‘financial’ items, which appeared to be a redundant theme in the acute care classification.

#### Face validity

Lay research partners’ impressions of relevance, appropriateness, and summarised comments are shown in Table 5. The COOP/WONCA, EQ-5D, MQoL-E, PcOS, and QoL-AD instruments had relevant thematic coverage and were shortlisted for potential recommendation. The remaining instruments were judged to be too focussed on specific outcome themes (Duke, SF-36, RHDS-OP-SF), or infeasible due to their length (TOPICS-CEP) and were not recommended for use by older people with frailty receiving emergency care.

**Table 5:** Instrument face validity informing reduction of a shortlist

Instrument	Theme relevance	Appropriate to setting	Summarised comments	Shortlist
COOP/WONCA	Moderate	Good	Superficial, accessible	Yes
Duke	Moderate	Poor	Focussed on self-esteem / existential	No
EuroQol	Moderate	Good	Superficial	Yes
MQoL-E	Good	Good	Appropriate themes for people with frailty Self-esteem less relevant	Yes
SF-36	Moderate	Poor	Focus on physical constructs Many activities beyond typical people with frailty	No
PcOS	Good	Good	Appropriate themes Very focused on existential	Yes
QoL-AD	Moderate	Moderate	Very specific to people with cognitive impairment	Yes
RHDS-OP-SF	Poor	Poor	Too specific to discharge period	No
TOPICS-CEP	Good	Poor	Appropriate level. Good coverage of physical and psychosocial Very long	No

#### Content validity

The QoL-AD instrument was excluded during the PPI Forum review stage based on it lacking content validity. The instrument was judged to be useful for people with mild cognitive impairment, but too focussed to be relevant to many people with emergency care needs. QoL-AD administration was by interview, and lay collaborators noted that while enabling access for some people, this could restrict accessibility for others, particularly those experiencing problems with insecurity either at home or in the healthcare setting.

The COOP/WONCA, EQ-5D, MQoL-E, and PcOS were judged to measure relevant themes and were suitably accessible to be considered as potential instruments for emergency care validation in future studies. Additional items are required to comprehensively measure the autonomy themes.

## Discussion

Four instruments which have been collected from older people with frailty in hospitals to measure health-related quality of life domains were recommended as having potential suitability for future studies emergency care settings: COOP/WONCA charts, EuroQol, McGill Quality of Life (Expanded), and Palliative care Outcome Scale. These co-produced recommendations were based on methodological quality, concept coverage, and face and content validity. Most studies used ‘very good’ quality methodologies for assessing measurement properties, and there was strong evidence for properties for most instruments. The identified instruments predominantly measured ‘Functioning’ domains.

### Strengths and limitations

This is the first systematic review to identify PROMs potentially suitable for older people with frailty and emergency care needs. Recommendations for potential suitability were formed through quality appraisal, construct mapping, and assessment of face and content validity with research collaborators. To ensure recommendations were grounded in a patient perspective, a co-production approach involved lay research partners at each stage of work. These partners had substantial knowledge of the population and setting and had advised many researchers on other ageing-related studies through the PPI Forum. However, they did not have expertise or specific training in clinical psychometrics, and therefore further work is indicated to confirm instrument measurement properties in this application.

Multiple databases and reference list hand-searches were included in the strategy, but we did not examine grey literature sources. Most citations were screened for eligibility by a single reviewer. However, there was strong interrater agreement for the 20% random sample of double-screened citations.

The COSMIN Risk of Bias checklist was used for quality appraisal, as all studies included some aspect of PROM development or validity evaluation. This checklist does require the researcher to apply judgment [33]. Appraisal was conducted by two reviewers (and supervised by the senior authors) who had expertise with the reference population and who were undertaking PhD research in PROMs. An instrument appraised in a study with ‘doubtful’ methodological quality was included in accordance with the COSMIN manual for systematic reviews [20]. The grade was reflected in the level of evidence summary and was considered during subsequent recommendation co-production. The review methodology sought evidence for instruments only from their use by older people living with frailty in hospital settings, and therefore did not fully consider the accumulated evidence for the measurement performance of these tools. Additional detailed evaluation of validity could use systematic reviews of evidence for each identified instrument [34].

### Implications for research and practice

Autonomy-themed healthcare outcomes (control, information, and safety) were poorly covered in these measures. Emergency care is unique in terms of its complexity, uncertainty, and consequent vulnerability [35, 36]. Autonomy outcomes may be absent from identified instruments due to the inclusion of studies with hospital inpatient participants, who may have been in more stable health with less uncertainty. Autonomy concepts are also harder to measure or correlate with existing outcomes data, and so traditionally may have been less attractive to consider alongside service metrics. Since autonomy has been identified as an outcome goal for older people’s acute care, further research into its measurement within PROMs is required.

Autonomy concepts are important and meaningful to patients and so should be measured. While these might usually be classified separately as aspects of care processes (and thus measured using PREMs), there is rationale for their consideration alongside PROMs among people who are living with frailty. People living with *severe* frailty have shorter life expectancy following acute care, and spend more of their time in healthcare settings [37]. They have similar needs to people receiving palliative care, for whom quality of life determinants also include having the knowledge and skills to be prepared for future healthcare interactions [38]. Indeed, ‘personal autonomy’ and ‘healthcare delivery’ are considered in the identified PROMs developed for people with palliative care needs [24, 39]. The merits of measurement using separate PROMs and PREMs or combined PROEMs is a matter for ongoing enquiry.

Instrument format is a key consideration for future research. Even people with advanced cognitive impairment have been able to self-report quality of life using PROM instruments [40]. Implementation programmes must ensure accessibility by accounting for factors known to affect completion including lower literacy and advancing age [41, 42].

The MQoL-E and PcOS instruments were notable for the relevance, range, and depth of information measured. However, these instruments used 21 and 10 items respectively, and both would require additional items to comprehensively measure autonomy themes. Longer instrument length presents a potential barrier to completion in emergency care settings by people living with frailty and requires evaluation in validation studies. There may be a trade-off between the desired data detail and the instrument accessibility which may feasibly be achieved.

EuroQol’s five-level version has been found to have better sensitivity and informativity than the three-level version, without posing problems with missing values [43, 44]. The responsiveness of EQ-5D has been identified as a property for further evaluation, and future studies comparing score changes against self-rated health have been recommended. EQ-5D scores should also be examined for discrepancies with preference values, which may foreseeably be encountered among people with frailty who have physical disability [45].

## Conclusion

Nine PROMs have been collected from older people living with frailty in acute care settings. The instruments were evaluated for emergency care suitability with lay and academic research collaborators, using construct mapping and assessment for face and content validity. The COOP/WONCA, EQ-5D, MQoL-E, and PcOS instruments were recommended as potentially suitable for validation studies with older people with frailty and emergency care needs. There was no ideal instrument which comprehensively measured all outcome goal themes for this population, and additional items are needed if autonomy domains are to be evaluated.

## Supplementary Information

**Additional file 1.** Detailed search strategy.

**Additional file 2.** Data extraction sheet.

**Additional file 3.** COSMIN scoring summary.

## Data Availability

The search strategy and data extraction sheets are provided as Supplementary Materials.

## Abbreviations

PROM: Person (or Patient) -Reported Outcome Measure
PREM: Person (or Patient) -Reported Experience Measure
MeSH: Medical Subject Heading
MQoL-E: McGill Quality of Life Questionnaire (Expanded)
PcOS: Palliative care Outcome Scale
QoL-AD: Quality of Life in Alzheimer’s Disease
RHDS-OP-SF: Readiness for Hospital Discharge Scale for Older People (Short Form)
TOPICS-CEP: The Older Persons and Informal Caregivers Survey composite endpoint
UK: United Kingdom
