# Supplementary material for: A systematic review and recommendations for prom instruments for older people with frailty in emergency care
Source: J Patient Rep Outcomes. 2022 Apr 1;6:30. doi: 10.1186/s41687-022-00438-x (PMC8975986; doi:10.1186/s41687-022-00438-x)
Supplement: Supplementary file 1 — Additional file 1. Detailed search strategy. [file 41687_2022_438_MOESM1_ESM.docx]

# A systematic review and recommendations for PROM instruments for older people with frailty in emergency care

# Supplementary material 1: search strategy

|  | *Population* |
| --- | --- |
| 1 | Health Services for the Aged/ or Geriatric Assessment/ or Frail Elderly/ or Frailty/ or Aging/ |
| 2 | (old* OR life OR aged OR elder* OR senior* OR geriatric* OR gerontol* OR veteran OR palliati* OR frail*).ti |
| 3 | 1 OR 2 |
|  | *Setting* |
| 4 | (hospital* OR ward OR emergency OR acute OR inpatient OR bed).ti,ab |
|  | *Intervention* |
| 5 | (PROM OR PROMs OR PRO OR PROs OR PREM OR PREMs OR patient reported outcome OR person reported outcome OR patient reported experience OR person reported experience).ti,ab |
| 6 | (measure* OR tool OR instrument OR questionnaire OR scale OR survey OR satisfaction).ti |
| 7 | 5 OR 6 |
|  | *Combine and limit results* |
| 8 | 3 AND 4 AND 7 |
| 9 | Apply limits to 8:   - 01 January 2010 to 24 June 2021 - English language |
